# Loss of *Snhg5* disrupts cell-cycle regulation without altering cystogenesis in a mouse model of polycystic kidney disease

**DOI:** 10.1038/s41598-026-35234-w

**Published:** 2026-01-08

**Authors:** Stephen D’Amico, Ujala Dar, Kara Eckberg, Ivan Weisser, Chandrema Hossain, Robert Bronstein, Karam Aboudehen

**Affiliations:** 1 Division of Nephrology & Hypertension, Department of Medicine, Stony Brook University, Stony Brook, NY USA; 2 Department of Medicine, University of Minnesota, Minneapolis, MN USA

**Keywords:** Cell biology, Genetics, Molecular biology, Diseases

## Abstract

Long non-coding RNAs (lncRNAs) regulate diverse cellular pathways and are increasingly linked to human disease. *Snhg5* is frequently described as a pathogenic lncRNA in many human diseases, including cancer. Our previous studies revealed that *Snhg5* is one of the most upregulated lncRNAs in multiple mouse models of polycystic kidney disease (PKD). Yet its role in renal biology and in autosomal dominant PKD (ADPKD) is not known. To elucidate the role of *Snhg5*, we generated a global *Snhg5*-null mouse. Homozygous animals were viable and displayed normal kidney morphology and function. RNA-sequencing of *Snhg5*-null kidneys and renal epithelial cells revealed common alterations in gene expression linked to cell cycle progression and DNA replication. At the molecular level, *Snhg5*-null cells showed increased sub-G1 and S/G2/M fractions, coinciding with depletion of ARPC5-a core ARP2/3 subunit-suggesting that reduced ARPC5 may contribute to this phenotype. To determine whether *Snhg5* upregulation is pathogenic in mouse PKD, we crossed *Snhg5*-null mice with a collecting duct-specific *Pkd1*-mutant mouse model. Loss of *Snhg5* did not attenuate cyst formation; if anything, disease severity was mildly but not significantly exacerbated. These findings indicate that *Snhg5* modulates cell-cycle control and is dispensable for kidney development and cystogenesis in collecting duct-derived cysts.

**Supplementary Information:**

The online version contains supplementary material available at 10.1038/s41598-026-35234-w.

## Introduction

Long non-coding RNAs (lncRNAs) are defined as non-coding transcripts longer than 200 nucleotides that lack a functional open reading frame^1^. Though non-coding by nature, they often share key features with protein-coding genes, including transcription by RNA polymerase II, exon–intron boundaries, alternative splicing, and polyadenylation^2,3^. Functionally, lncRNAs operate at nearly every level of gene expression from modulating chromatin accessibility and epigenetics to influencing transcription, splicing, mRNA stability, and translation^4,5^. A growing number of lncRNAs have been directly implicated in human disease, particularly in cancer^6^. For instance, *PVT1* (Plasmacytoma Variant Translocation 1) is often aberrantly expressed in various carcinomas including esophageal, nasopharyngeal, gastric, colorectal, and pancreatic cancer^7^. Furthermore, its genomic position coincides with a known translocation breakpoint in myeloid and lymphoid neoplasms^8,9^. *MALAT1* (Metastasis Associated Lung Adenocarcinoma Transcript 1) has been proposed to mediate chemotherapy and drug resistance due to its ubiquitous expression and participation in diverse cellular processes such as epithelial-to-mesenchymal transition (EMT), autophagy, and DNA damage repair^10^.

*SNHG5* (small nucleolar RNA host gene 5) is a relatively well-characterized lncRNA that has been implicated in multiple malignancies, including colorectal cancer, gastric cancer, glioma, and hepatocellular carcinoma^11–16^. *SNHG5* appears capable of functioning as either a tumor promoter or inhibitor depending on the cell type, indicative of its context dependency and tissue-specificity. Beyond cancer, mouse *Snhg5* has also been implicated in diabetic nephropathy^17^ and sepsis-induced acute kidney injury (AKI), suggesting broader roles in cell growth, homeostasis, and disease^18^. The increasing number of lncRNAs now recognized as critical disease-associated genes has not only reshaped our understanding of non-coding RNA biology but also stimulated broader investigations into how other lncRNAs may fit into the paradigm of human development and disease.

Autosomal dominant polycystic kidney disease (ADPKD) is a genetic disorder primarily caused by germline mutations in the *Pkd1* and *Pkd2* genes^19^. Recent estimates place ADPKD prevalence rate at approximately 1 in 1000 people^20^. The disease is characterized by the progressive formation of numerous fluid-filled cysts, kidney dysfunction, and eventual progression to end-stage renal disease (ESRD)^21,22^. ADPKD is a significant health burden and there is an urgent unmet need for therapeutic intervention. We previously demonstrated that the lncRNAs *Hoxb3os* and *Pvt1* are modulators of cystogenesis^23–25^. Ablation of *Hoxb3os* in mouse ADPKD exacerbated cyst formation, indicating a protective role, whereas shRNA-mediated knockdown of *Pvt1* in metanephric organ culture attenuated cyst growth, implicating *Pvt1* as a promoter of cystogenesis. We observed *Snhg5* to be among the top-upregulated lncRNAs in *Pkd1-* and *Pkd2-* mutant mice. Despite its recurrent dysregulation in disease, no global or tissue-specific in vivo knockout models of *Snhg5* have been reported, rendering its role in normal development and in mouse PKD unknown.

To address this knowledge gap, we generated a global *Snhg5*-null mouse to characterize its role in normal kidney function and PKD pathogenesis. Our study yielded four key observations: (1) *Snhg5* expression is dysregulated in both human and mouse models of ADPKD; (2) *Snhg5*-null kidneys are phenotypically normal under baseline conditions; (3) Deletion of *Snhg5* resulted in late stage cell-cycle perturbations and downregulation of ARPC5 protein; and (4) *Snhg5* is dispensable for cyst formation in a collecting duct (CD)-specific *Pkd1*-mutant mouse model. Together, these findings provide a comprehensive in vivo assessment of *Snhg5* in the kidney and suggest that, despite its dysregulation in ADPKD, it does not affect disease progression in a CD mouse model.

## Results

### Characterization of *Snhg5* in the mouse

**Fig. 1:**
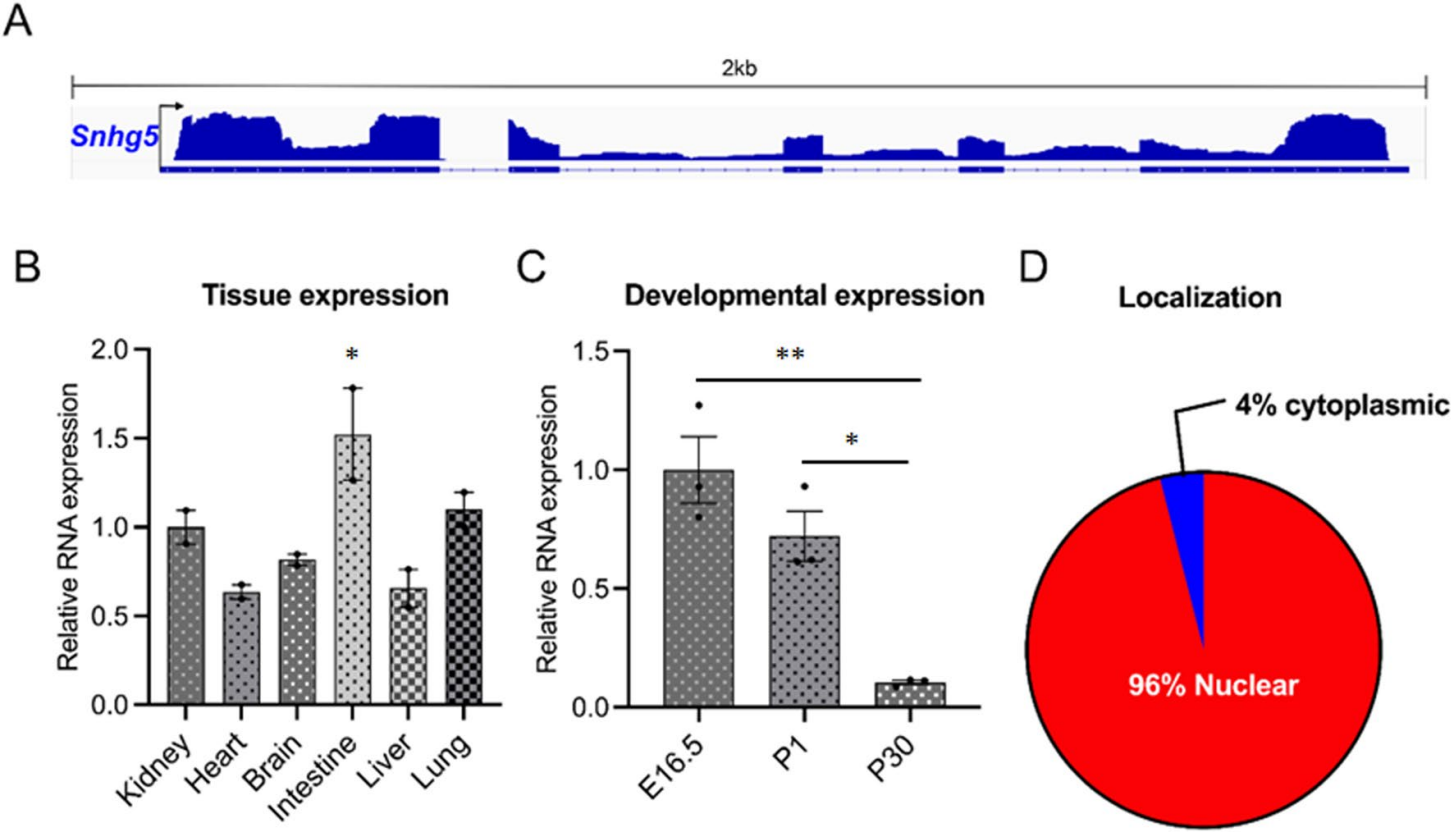
Characterization of lncRNA *Snhg5* expression in the mouse. (**A**) Schematic diagram showing the 1.9 kb *Snhg5* locus on mouse chromosome 9. Thick and thin blue bars designate exons and introns, respectively. RNA sequencing reads (top) from mouse kidney (P20) are displayed in blue. (**B**) qRT-PCR showing ubiquitous expression of *Snhg5* in various tissues from adult C57Bl/6 mice (P30). Error bars represent SD. (**C**) qRT-PCR showing that the expression of *Snhg5* in the kidney is developmentally regulated decreasing from E16.5 to P30. Error bars represent SD. (**D**) qRT-PCR showing that *Snhg5* in the mouse kidney (P30) is predominately expressed and localized to the nucleus (96%) vs. the cytoplasm (4%).

Given reports that *Snhg5* is an active participant in multiple human cancers with potential roles in kidney disease, we set out to functionally characterize *Snhg5* in the mouse kidney. The murine *Snhg5* locus, located on chromosome 9, is ~ 1.9 kb in length with five exons encoding a 1084-nt transcript (Fig. 1A). Total RNA from various tissues of adult wild-type C57Bl/6 mice was analyzed by qRT-PCR. Expression analysis revealed that *Snhg5* is ubiquitously expressed with the highest levels detected in the intestines among the tissues examined (Fig. 1B). Expression was also analyzed at different stages of kidney development: at embryonic day 16.5 (E16.5), in the newborn at post-natal day 1 (P1), and in the early adult mouse at P30. *Snhg5* expression decreased by > 90% from E16.5 to P30, suggesting it is a developmentally regulated lncRNA (Fig. 1C). Cellular fractionation of the adult mouse kidney (P30) and subsequent qRT-PCR analysis demonstrated that the vast majority (96%) of *Snhg5* transcripts are localized to the nuclear compartment, with only 4% detected in the cytoplasm (Fig. 1D).

### *Snhg5 e*xpression is dysregulated in human ADPKD and mouse PKD

**Fig. 2:**
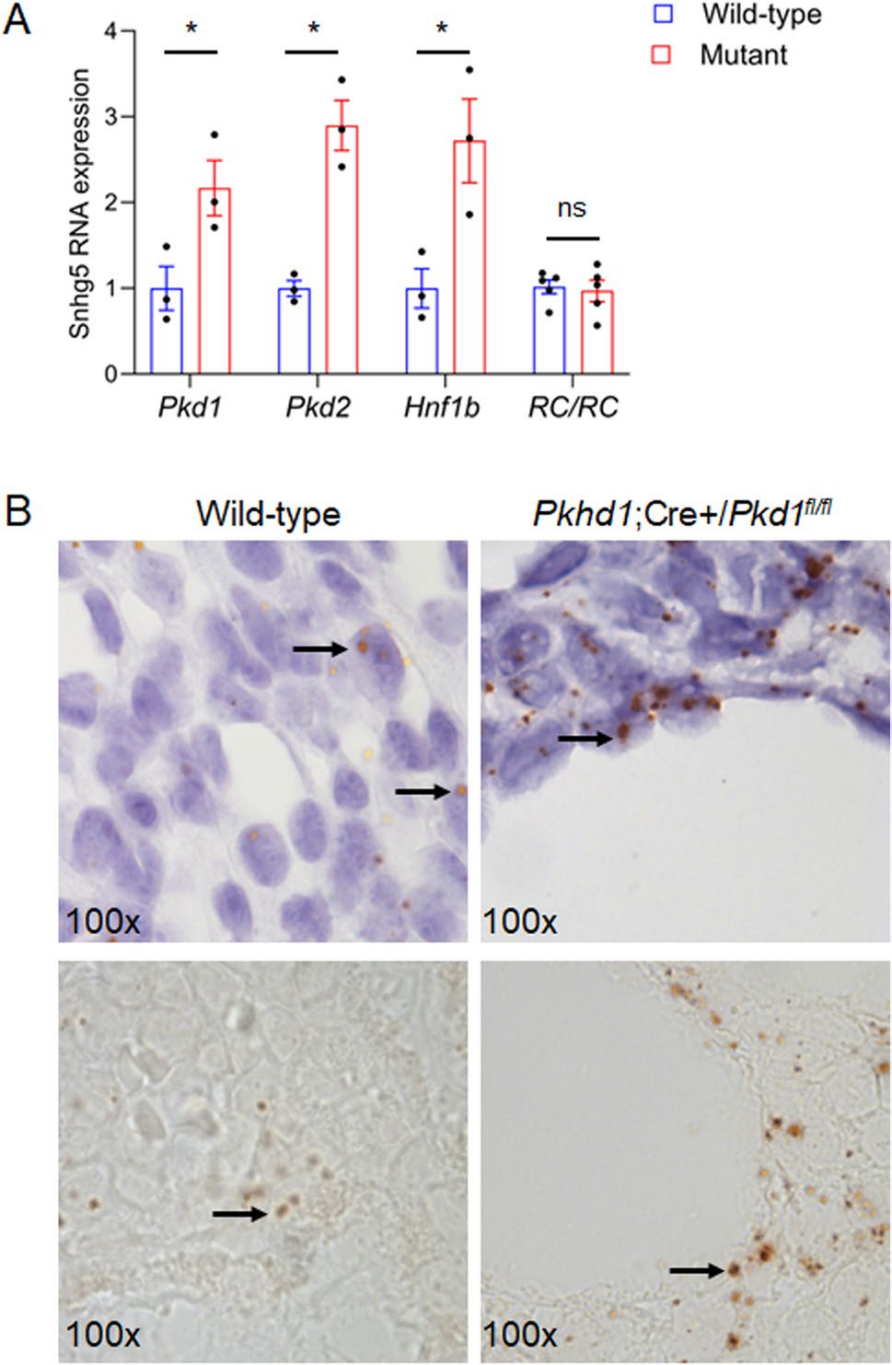
*Snhg5* expression is upregulated in rapidly progressing mouse models of PKD. (**A**) qRT-PCR showing the expression of *Snhg5* lncRNA in *Pkhd1*-Cre;*Pkd1*^fl/fl^ (P10, *n* = 3), *Pkhd1*-Cre;*Pkd2*^fl/fl^ (P21, *n* = 3), *Pkhd1*-Cre;*Hnf1b*^fl/fl^ (P35, *n* = 3), and *Pkd1*^RC/RC^ (*n* = 5) mouse models of cystogenesis relative to wild-type controls. Data are normalized to U6 or b-actin. Error bars represent SD. (**B**) RNAscope demonstrating the localization of *Snhg5* RNA in the kidneys of wild-type (left) and *Pkhd1*-Cre;*Pkd1*^fl/fl^ (right) mice. Sections are shown with (top) and without (bottom) hematoxylin counterstain. Black arrows designate positive nuclear staining.

RNA-seq data from our previous study revealed that *Snhg5* is upregulated in cystic kidneys derived from both *Pkd1*- and *Pkd2*-null mice^23^. We confirmed this by qRT-PCR in which *Snhg5* RNA was elevated by approximately 2–3-fold in cystic kidneys from *Pkd1*-, *Pkd2-*, as well as *Hnf1b*-mutant mice (another cystic mouse model), with samples analyzed at P10 (for *Pkd1*), P21 (for *Pkd2*), and P35 (for *Hnf1b)* (Fig. 2A). We also examined *Snhg5* expression in cystic kidneys from *Pkd1*^RC/RC^ mice at 6 months. This hypomorphic mouse model is characterized by a slow-progressing and less severe cystic phenotype that more closely recapitulates the disease course in human ADPKD^26^. In contrast, *Snhg5* expression was unchanged in this mouse model (Fig. 2A). Next, to define the spatial expression of *Snhg5* in the mouse kidney, we performed RNAscope on wild-type, *Pkd1*-null, and *Hnf1b-*null sectioned kidneys. Two main observations emerged: (1) *Snhg5* expression was ubiquitously expressed in nuclei across all nephron segments and (2) *Snhg5* expression was markedly higher in cystic as well as adjacent non-cystic epithelial cells of *Pkd1*- and *Hnf1b*-null kidneys compared with wild-type controls (Fig. 2B, Supplementary Fig. S1).

*Snhg5* is an evolutionarily conserved lncRNA. Its human ortholog, *SNHG5*, resides on chromosome 6 and encodes a 524-nt transcript^27^. To determine whether *SNHG5* is similarly dysregulated in human ADPKD, we quantified its expression in nephrectomy specimens from ADPKD patients and matched adjacent normal kidney tissue. In contrast to the upregulation seen in rapidly progressing mouse PKD, *SNHG5* RNA was reduced by > 90% in human ADPKD samples (Supplementary Fig. S1). The downregulation of *SNHG5* is corroborated by published studies of single-nucleus RNA-seq (snRNA-seq)^28^ where both the average transcript level and the fraction of *SNHG5*-positive cells declined across all cell types in cystic kidneys (Supplementary Fig. S1). Combined with our findings in the *Pkd1*^RC/RC^ mouse model, these data suggest that while *Snhg5/SNHG5* dysregulation is a shared molecular feature of cystogenesis, its upregulation may be transient in rapid-progressing PKD.

### *Snhg5*-null mice are phenotypically normal

**Fig. 3:**
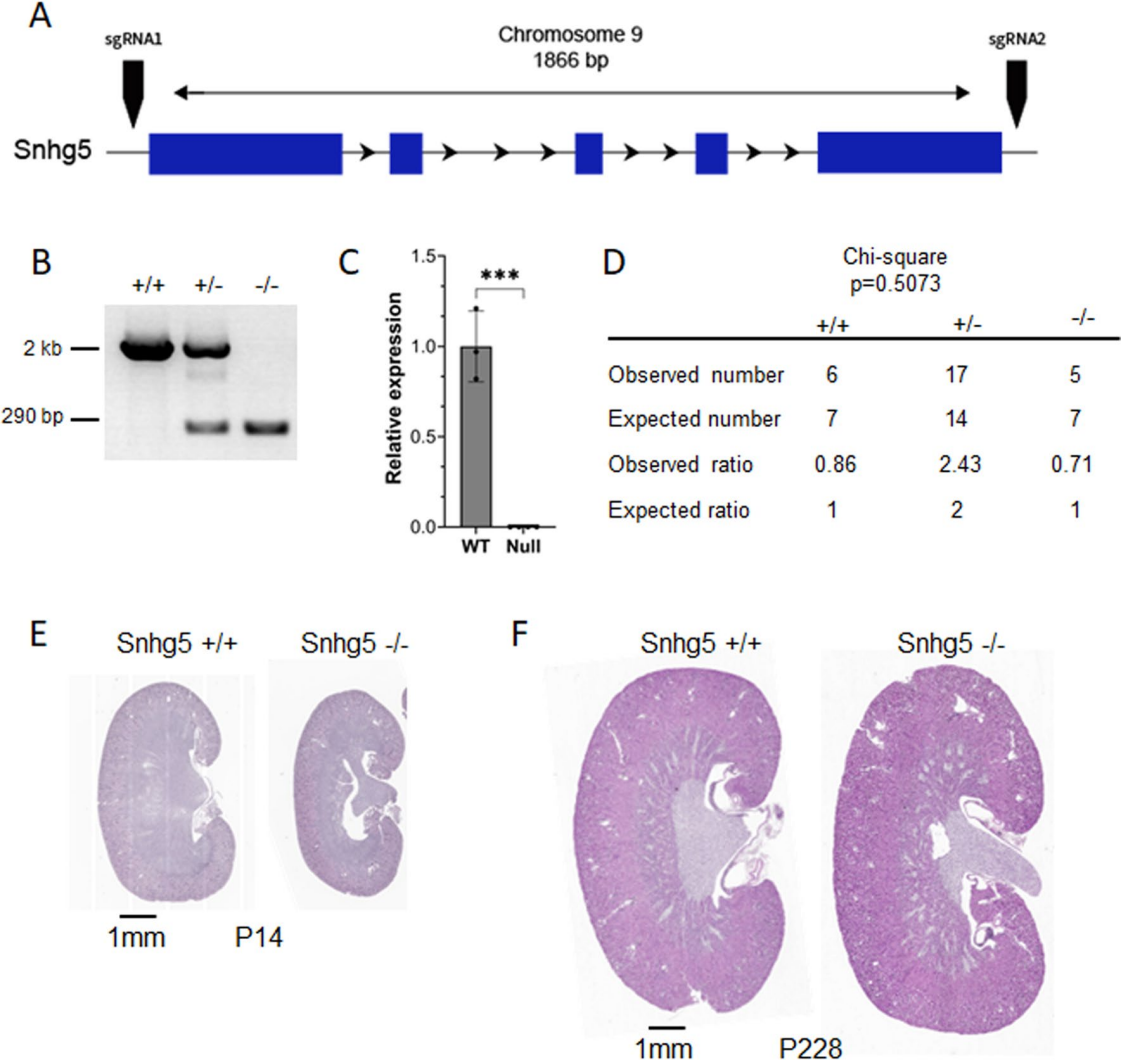
Generation of a global *Snhg5* knockout mouse model. (**A**) Schematic of CRISPR/Cas9-mediated strategy to generate a global *Snhg5*-null mouse. Exons and introns are shown as thick (blue) and thin (black) lines, respectively. Horizontal arrows indicate the direction of transcription and vertical arrows denote sgRNA target sites. (**B**) PCR products amplified from genomic DNA extracted from mice (P14) of the indicated genotypes. Genotyping primers flank the outside of the deleted region. High molecular weight band (~ 2 kb) represents the intact wild-type allele, and the lower band (~ 290 bp) shows the recombined null allele. Primer sequences are provided in Table 2. (**C**) qRT-PCR on RNA extracted from mouse kidneys (P14) of the indicated genotypes confirming loss of *Snhg5* expression in *Snhg5*-null mice. (**D**) Chi-square test (*p* = 0.5073) showing the observed and expected numbers and ratios of the indicated mouse genotypes (P20, *n* = 28). (**E**) Representative full kidney sections stained with H&E from wild-type (+/+) and *Snhg5*-null (−/−) mice at P14. Scale bar = 1 mm. (**F**) Stained kidney sections at P228 as described in panel E.

**Fig. 4:**
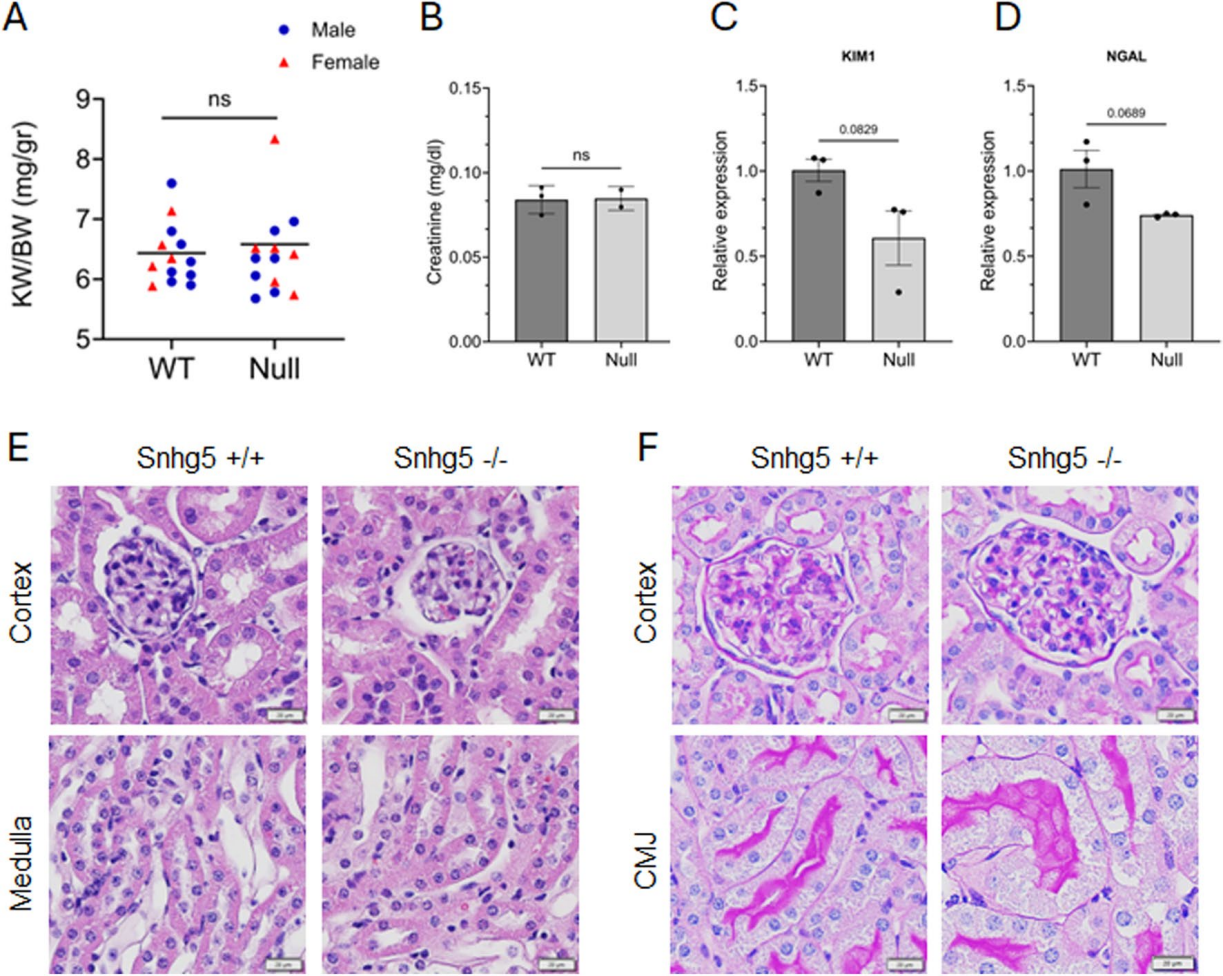
*Snhg5* null mice exhibit normal kidney morphology and function. (**A**) Kidney weight-to-body weight ratio (KW/BW) of wild-type (*n* = 13) and *Snhg5*-null mice (*n* = 13) at P14. Blue circles represent males, and red triangles are female mice. Horizontal black lines denote the mean. (**B**) Serum creatinine measurements (mg/dL) of a subset of wild-type (*n* = 3) and *Snhg5*-null mice (*n* = 2) at P14. Error bars represent SD. (**C**) Expression levels of the kidney injury marker *Havcr1* (KIM1) measured by qRT-PCR on RNA extracted from wild-type and *Snhg5*-null mice at P228. Error bars represent SD. (**D**) Expression of *Lcn2* (NGAL) as described in panel C. (**E**) Representative H&E staining of kidney sections from the cortex and medulla of wild-type (+/+) and *Snhg5*-null (−/−) mice at P228. Scale bar = 20 μm. (**F**) Representative Periodic acid-Schiff (PAS) staining of kidney sections from the cortex and corticomedullary junction (CMJ) of wild-type (+/+) and *Snhg5*-null (−/−) mice at P228. Scale bar = 20 μm.

Nearly all studies on *Snhg5* function utilize shRNA or other transient knockdown strategies. To generate a loss-of-function model, we deleted the entire 1.9 kb *Snhg5* locus in C57Bl/6 zygotes using CRISPR/Cas9-mediated gene editing (Fig. 3A). Cas9 protein and two single guide RNAs (sgRNAs) were injected into mouse zygotes and then implanted into pseudo-pregnant foster mothers. Successful deletion of the *Snhg5* genomic locus was confirmed via PCR on genomic DNA from 14-day-old mice (Fig. 3B). We next confirmed elimination of the *Snhg5* transcript via qRT-PCR on total RNA extracted from the kidneys of mice at P14. *Snhg5*-null mice had virtually undetectable *Snhg5* RNA levels compared to wild-type controls (Fig. 3C). *Snhg5*-null mice were born at Mendelian ratios and lived a normal lifespan (Fig. 3D). Next, we conducted histological, morphological and functional examinations at P14 and P228. Irrespective of age, *Snhg5*-null kidneys were of comparable size to their wild-type counterparts and exhibited normal cortical and medullary organization with clearly defined corticomedullary junctions (Fig. 3E and F). Furthermore, compared to wild-type controls, *Snhg5* null mice displayed a similar kidney weight-to-body weight (KW/BW) ratio, both as a unified whole and when stratified by sex (Fig. 4A). Staining with hematoxylin and eosin (H&E) revealed normal cortical and medullary morphology in *Snhg5*-null kidneys (Fig. 4E). Similarly, periodic acid–Schiff (PAS) staining showed intact tubular and glomerular structures without evidence of fibrosis, inflammation, or brush border effacement within proximal tubules (Fig. 4F). Functionally, serum creatinine levels remained within normal limits, while expression of the classical renal injury markers *Havcr1* (KIM1) and *Lcn2* (NGAL) was slightly, but not significantly reduced relative to wild-type mice (Fig. 4B–D). Taken together, these findings suggest that *Snhg5* is likely dispensable for normal kidney development, morphology, and function.

### *Snhg5* mediates cell cycle progression

**Fig. 5:**
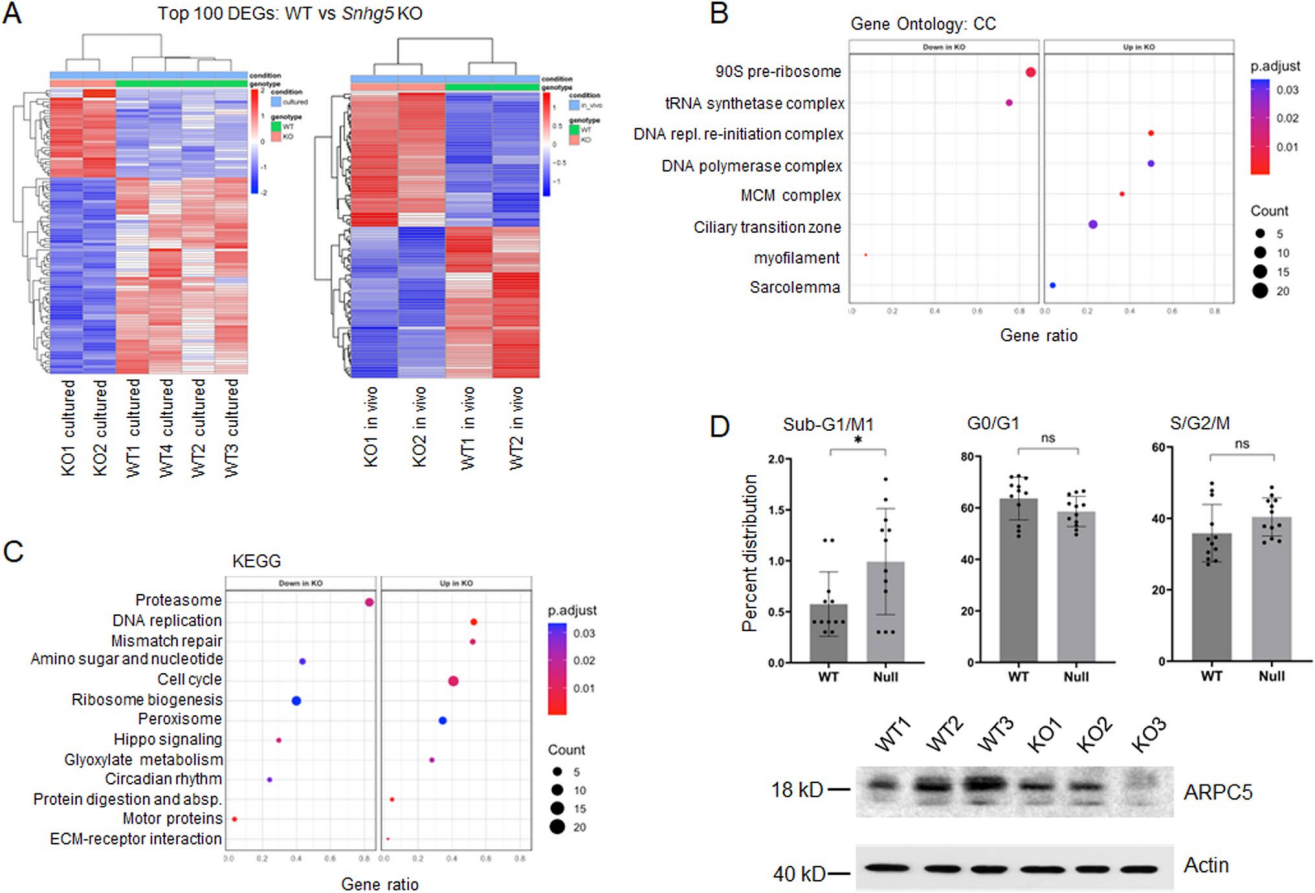
Deletion of *Snhg5* causes dysregulated cell cycle pathways. (**A**) Heatmaps depicting the top 100 differentially expressed genes in (left) isogenic *Snhg5*-null (*n* = 2) vs. wild-type (*n* = 4) mIMCD3 cells, and (right) *Snhg5*-null (*n* = 2) vs. wild-type (*n* = 2) kidneys. Rows represent differentially expressed genes. Columns represent biological replicates (*n* = 10). Expression values are shown as Z-scores of normalized CPM, with red indicating higher and blue indicating lower expression relative to the mean across samples. (**B**) Gene ontology (cellular component) derived from integrative differential expression analysis of wild-type vs. *Snhg5*-null mIMCD3 cells and kidneys. (**C**) Gene set enrichment (GSEA) derived from integrative differential expression analysis of wild-type vs. *Snhg5*-null mIMCD3 cells and kidneys. (**D**) Top, cell cycle profile of isogenic wild-type (*n* = 4) and *Snhg5*-null (*n* = 4) mIMCD3 cells showing distribution across sub-G1/M1 (*p* = 0.027, left), G0/G1 (*p* = 0.099, middle), and S G2/M (*p* = 0.117, right) phases. Mean values are shown. Error bars represent SD. Bottom, Western blot analysis confirming the downregulation of ARPC5 protein in *Snhg5*-null mIMCD3 cells relative to wild-type cells. Actin is a loading control.

Given that *Snhg5* is predominantly nuclear and its function remains undefined in both the in vivo mouse kidney and in vitro renal cells, we asked whether loss of *Snhg5* perturbs transcriptional networks under baseline conditions. To accomplish this, we performed whole transcriptome RNA-seq on *Snhg5*-null and wild-type kidneys, as well as on multiple isogenic *Snhg5*-null mIMCD3 cell lines generated with CRISPR/Cas9. *Snhg5*-null kidneys displayed only a total of 139 differentially expressed genes, consisting of 65 up-regulated and 74 down-regulated transcripts (Fig. 5A, Supplemental Fig. S2). The downregulation of select genes was validated within independent *Snhg5* KO kidney samples using qRT-PCR (Supplemental Fig. S2). Pathway analysis of the in vivo dataset revealed broadly enriched categories related to transcription, regulation of gene expression, and lipid metabolism, consistent with the modest magnitude of transcriptional change (Supplemental Fig. S2). In contrast, *Snhg5*-null mIMCD3 cells showed a more robust response, with a total of 500 differentially expressed genes, comprising 321 up-regulated and 179 down-regulated transcripts. Pathway enrichment of mIMCD3 cells highlighted changes in cell proliferation, apoptosis and migration, as well as perturbation of cancer-associated MAPK and PI3K-Akt signaling, reflecting more defined effects upon *Snhg5* loss (Fig. 5A, Supplemental Fig. S2). To further strengthen the analysis, we performed a combined in vitro and in vivo analysis and identified a total of 1,524 genes that were concordantly dysregulated across the combined datasets, comprising 815 up-regulated and 709 down-regulated genes (Supplemental Fig. S3). Across multiple analyses of the integrated dataset, the top dysregulated pathways upon *Snhg5* deletion included upregulation of cell cycle and DNA replication (Fig. 5B and C, Supplemental Fig. S3), suggesting that *Snhg5* regulates cell cycle progression.

Functionally, cell cycle profiling through PI-FACS analysis showed a significant increase in the sub-G1/M1 fraction in *Snhg5*-null clones compared to wild-type controls (Fig. 5D). While G0/G1 and S/G2/M distributions did not reach statistical significance, there was a trend toward decreased G0/G1, and increased S/G2/M compared to control, consistent with impaired late-stage cell-cycle progression in *Snhg5*-null cells. To identify candidate effector protein(s) responsible for the cell cycle distribution in *Snhg5*-null cells, we re-examined our in vitro RNA seq data and noted decreased expression of the *Arpc5* gene, a core subunit of the ARP2/3 complex, which is required for nucleating branched actin filaments during cell division. Disruption of ARP2/3 function impairs actomyosin ring assembly leading to cytokinesis failure and M phase arrest^29–33^. Western blot analysis confirmed that ARPC5 expression was also diminished at the protein level in *Snhg5*-null mIMCD3 cells relative to wild-type controls (Fig. 5D). These data suggest that *Snhg5* may help maintain proper cell-cycle integrity, potentially through sustaining *Arpc5* expression.

### *Snhg5* is dispensable for cystogenesis in PKD1 mutant mice

**Fig. 6:**
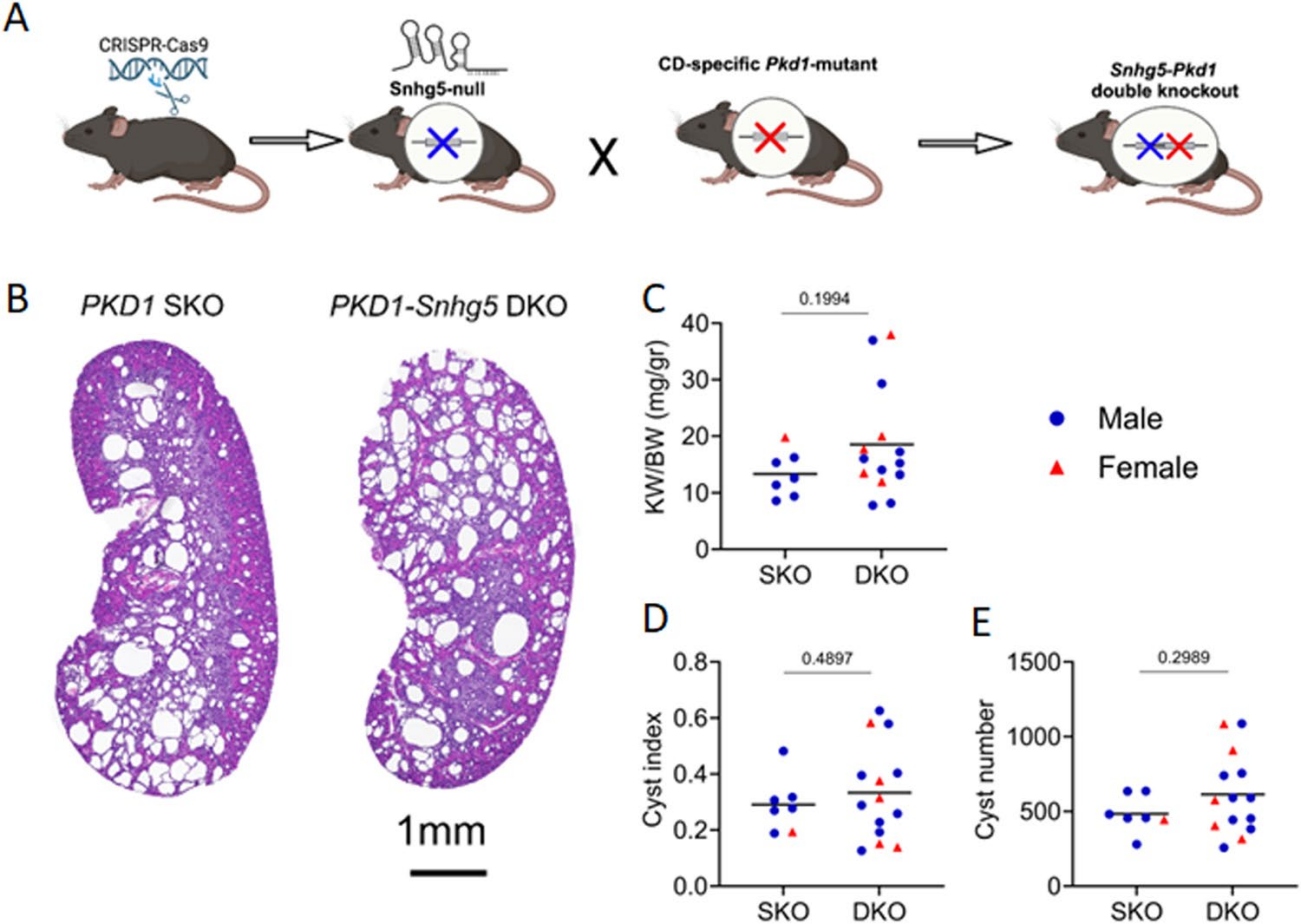
*Snhg5* is dispensable for cystogenesis in a collecting duct-specific PKD1 mouse model. (**A**) Schematic diagram detailing the breeding strategy used for the generation of *Pkd1*-SKO and *Pkd1-Snhg5*-DKO mice. (**B**) Representative H&E staining of full kidney sections from 10-day-old *Pkd1*-SKO (*n* = 7) and *Pkd1-Snhg5*-DKO (*n* = 14) mice. (**C**)–(**E**) KW/BW ratios, cyst index, and cyst number show a non-significant trend towards increased cystogenesis in DKO mice, independent of sex. Blue circles represent male mice, and red triangles are female mice. Black lines denote the mean.

Because genetic loss of *Snhg5* disrupted transcriptional programs that control cell-cycle progression in collecting duct cells and kidneys, and since unchecked proliferation is a hallmark of ADPKD, we next asked whether deleting *Snhg5* could modify cyst growth *in vivo.* To do so, we employed the well-established *Pkhd1-Cre; Pkd1*^*fl/lf*^ mouse model^34^, in which *Cre* recombinase excises *Pkd1* selectively in the collecting ducts (CD). Consequently, every cyst that forms is unambiguously of CD origin allowing us to focus specifically on the contribution of *Snhg5* to CD-derived cystogenesis. We first bred *Snhg5*^+/−^ mice with *Pkhd1*/*Cre*; *Pkd1*^fl/+^ mice and intercrossed their progeny to generate *Pkd1* single knockout (SKO) (*Pkhd1*/*Cre*+; *Pkd1*^fl/fl^; *Snhg5*^+/+^) and *Pkd1*-*Snhg5* double knockout (DKO) (*Pkhd1*/*Cre*+; *Pkd1*^fl/fl^; *Snhg5*^−/−^) mice (Fig. 6A). The kidneys of these mice were harvested at P10 and analyzed by H&E. Cystic burden was quantified using three independent metrics: (1) kidney weight-to-body weight ratio (KW/BW); (2) cyst index, calculated as cystic area divided by non-cystic parenchyma; and (3) absolute cyst number. Across all three readouts, DKO kidneys exhibited a mild exacerbation of cystic disease relative to SKO controls as the mean KW/BW, cyst index, and cyst counts were each modestly higher in the DKO cohort (Fig. 6B–E). Nevertheless, none of these differences reached statistical significance whether analyzed collectively or separated by sex. Taken together, the results show that upregulation of *Snhg5* in murine PKD is not required for cyst initiation or expansion in the CD *Pkd1* model. Thus, despite its strong early transcriptional induction and its influence on mitotic gene networks, *Snhg5* is functionally dispensable for PKD pathogenesis in this setting.

## Discussion

lncRNAs are emerging as pivotal regulators of gene expression^3^, chromatin organization^35^, and cellular homeostasis^36^, yet for the majority of lncRNAs, their physiological roles remain poorly defined. This knowledge gap is especially evident in ADPKD, in which the pathogenesis is driven by cyst-forming mutations in *PKD1* or *PKD2*^*21*^, and is characterized by unchecked epithelial proliferation^37^. While many lncRNAs are transcriptionally dysregulated in cystic kidneys^23^, few have been tested functionally in vivo^38^, leaving open the question of whether their altered expression is a cause or a consequence of disease. In our previous work, we identified lncRNAs that are consistently dysregulated in PKD, including several that are downregulated^23^. Notably, *Hoxb3os* was among the top downregulated lncRNAs across multiple mouse models. Functional studies revealed that *Hoxb3os* deletion exacerbates cyst growth by enhancing mTORC2 signaling in vivo^25^. In contrast, *Snhg5* emerged as one of the most strongly upregulated lncRNAs in mouse PKD. Despite this significant upregulation, the role of *Snhg5* in kidney development and cystogenesis is completely unknown. Here we combine global *Snhg5* knockout mice, CRISPR-edited renal cell lines, and a collecting duct (CD)-specific *Pkd1* mouse model to define the in vivo and in vitro functions of *Snhg5*, determine its molecular impact, and assess its requirement for normal kidney development and cyst formation/progression in mouse PKD.

Our initial *Snhg5* expression profiling revealed major findings. First, unlike many lncRNAs that are specific to a handful of tissues^35^, *Snhg5* transcripts are detectable in every adult mouse organ we examined indicating that its function is not restricted to the mammalian kidney. Second, *Snhg5* is developmentally regulated, wherein transcript levels decreased by > 90% from E16.5 to P30, a period when proliferative nephrogenic zones give way to quiescent mature nephrons. This pattern suggests that *Snhg5* may act most prominently during phases of rapid cell division. Third, subcellular fractionation revealed that 96% of renal *Snhg5* resides in the nucleus. Although published studies have reported mixed nuclear or cytoplasmic localization for *SNHG5* in human cell lines^39,40^, our data establish a predominantly nuclear pattern in vivo, pointing toward roles in mRNA processing, transcriptional regulation, or chromatin remodeling rather than translation control. Finally, in humans, *SNHG5* expression is decreased in the kidneys of patients with ADPKD^28^. This is in contrast to the upregulation of *Snhg5* in mouse models of PKD. The difference in expression between human ADPKD and mouse PKD may be explained by the timing and course of the disease. ADPKD in humans takes decades to manifest with a median age for end-stage renal disease (ESRD) at 58.1 years for *PKD1* mutations and 79.7 years for *PKD2*^41^. Consequently, human specimens are collected from ADPKD patients with advanced disease after nephrectomy. By contrast, our analyses in mouse models were performed between postnatal days 10 and 35, when cyst expansion and epithelial proliferation are most active. Supporting this interpretation, we found that *Snhg5* expression was unchanged in the slow-progressing cystic *Pkd1*^RC/RC^ mouse model at 6 months of age. This model more closely recapitulates the chronic disease course seen in human ADPKD. It is possible that expression of *Snhg5* in a slow-progressing cystogenesis model at terminal disease stage would parallel the downregulation in the human. Taken together, this suggests that the apparent species divergence likely reflects differences in disease stage rather than fundamentally distinct regulation.

To determine whether *Snhg5* affects normal development of the mammalian kidney, we generated an in vivo global knockout of *Snhg5* in the mouse. Overall, *Snhg5*-null mice were phenotypically normal. The lack of an apparent kidney phenotype was confirmed upon histological examination; however, we cannot rule out the effect of *Snhg5* removal on the morphology and functionality of other organ systems, which is outside the scope of the present study. It is conceivable that *Snhg5* operates to fine-tune developmental processes rather than act as a true master regulator. This seems to be the case as we were able to detect subtle alterations at the level of gene expression by RNA seq analysis of *Snhg5*-null mIMCD3 cells and kidneys. Combined pathway analysis from in vivo and in vitro *Snhg5*-null models revealed perturbations in several pathways, with the most consistent being upregulation of cell-cycle and DNA-replication. These findings were partially supported by FACS analysis, which showed *Snhg5*-null cells displaying reduced G0/G1 and increased S/G2/M and sub-G1/M1 fractions, indicative of altered cell-cycle progression. A potential mechanistic framework that explains this distribution centers on downregulation of ARPC5, a structural component of the ARP2/3 complex that nucleates branched actin filaments essential for spindle positioning, contractile-ring formation, and successful cytokinesis^31,33^. Inhibiting ARP2/3 complex slows or stalls these processes, disrupting late cell-cycle events such as cytokinesis^29^. Alternatively, the downregulation of ARPC5 may reflect an uncoupling between normal cell cycle progression and cytoskeletal dynamics induced upon *Snhg5* loss. In this scenario, increased proliferative signaling reduces cellular prioritization for ARPC5 resulting in transcriptional repression of *Arpc5*. Whether *Snhg5* stabilizes *Arpc5* mRNA, recruits transcription factors to its promoter, or modulates chromatin accessibility at the *Arpc5* locus remains to be determined.

Our genetic crosses show that removing *Snhg5* in a CD-*Pkd1* background had, at most, a subtle effect on cyst burden. Across three quantitative readouts- KW/BW, cyst index, and cyst number-double-knockout (DKO) animals trended slightly higher than single-knockout (SKO) controls, yet none of the differences reached statistical significance. These data indicate that, within the confines of this CD-restricted cystic model and the early post-natal window examined, *Snhg5* is not a rate-limiting determinant of cyst initiation or short-term expansion. Several points help frame this negative result. First, in human ADPKD, only a subset of cysts arises from collecting-duct (CD) epithelia^42^, whereas our *Pkhd1*-*Cre* mouse model deletes *Pkd1* exclusively in CD cells, resulting in the rapid development of CD-derived cysts. Consequently, the model enables evaluation of *Snhg5’s* role only in CD cystogenesis and may overlook effects it might exert in cysts that originate from proximal tubule, loop of Henle, or other nephron segments. Second, because epithelial proliferation in this rapid cystic model is already near its maximum, a lncRNA that merely extends M-phase slightly, as our cell cycle assays suggest for *Snhg5*, would be unlikely to exert a measurable influence on overall cyst burden. Third, transcriptional redundancy is common among lncRNAs^43^; paralogous transcripts or compensatory pathways could mask the loss of *Snhg5 in vivo*. Finally, our evaluation was limited to a single, early time point. As cysts enlarge, mechanical stress, hypoxia, and inflammatory signals accumulate, conditions that might unmask *Snhg5* dependence at later stages. These caveats notwithstanding, the absence of a strong phenotype argues against *Snhg5* being a primary driver of cystogenesis in this setting. Future work should test *Snhg5* in alternative models, such as *Pkd2*, or inducible adult cystic models.

In summary, our study shows that despite being one of the top upregulated lncRNAs in mouse PKD, *Snhg5* loss does not alter cyst initiation or early expansion in a CD *Pkd1*-mutant mouse model. We show that *Snhg5* is ubiquitously expressed, developmentally regulated, and predominantly nuclear. Loss of *Snhg5* disrupts a mitotic gene network centered on *Arpc5*, but is nonetheless dispensable for baseline renal development, function, and CD-derived cystogenesis. The opposing direction of *Snhg5* dysregulation in mouse and human kidneys highlights the complexity of lncRNA control across species and disease stages, reinforcing the need to test both loss- and gain-of-function alleles in more than one experimental context before designating any lncRNA as a therapeutic target.

## Materials and methods

### Animal studies

All procedures related to animals were reviewed and approved by the Institutional Animal Care and Use Committee at Stony Brook University and conducted in accordance with relevant guidelines and regulations. The animal research was carried out in accordance with the ARRIVE guidelines. Efforts were made to minimize the number of animals used and their suffering to the greatest extent possible, which was in line with the current animal welfare regulations Table 1.

**Table 1:** RT-PCR primers.

Gene target	Forward Primer (5′–3′)	Reverse Primer (5′–3′)
*Actb*	CTCTGGCTCCTACACCATGAAGA	GTAAAACGCAGCTCAGTAACAGTCCG
*Cish*	CCCTGTCCAGGCAGAGAATG	TAGGAATGTACCCTCCGGCA
*Clec2d*	CTTGCCCGCAAAACTGGATT	GACTCTCTGTGCAGGCCAAT
*Ints6*	TCAGCCTCGAAGGTTGCATA	TCAAGAGTGGAGAGGCACAC
*Snhg5*	TCCGAAGGTACTAGAGTCACC	CTCTCAGATGCTTTGGGCTCC
*SNHG5*	TGTCTTCAGTGGCACAGTGGA	CTGTTAAAAGTGTCAGGTTT
*U6*	CTCGCTTCGGCAGCACA	AACGCTTCACGAATTTGCGT
*Havcr1 (KIM1)*	AGCAGTCGGTACAACTTAAAGG	AGAGTCTCTATCGTCAAGGACA
*Lcn2 (NGAL)*	GCAGGTGGTACGTTGTGGG	CTCTTGTAGCTCATAGATGGTGC

**Table 2:** Genotyping primers.

Target	Forward Primer (5′–3′)	Reverse Primer (5′–3′)
*Snhg5* (internal)	TCCGAAGGTACTAGAGTCACC	CTCTCAGATGCTTTGGGCTCC
*Snhg5* (flanking)	GACAAGTTCTCAGGGAAACC	GCCCCGGAATGGCCTGTAAA
*Pkd1l* ^*fl/fl*^	CCGCTGTGTCTCAGTGTCTG	CAAGAGGGCTTTTCTTGCTG
*Pkd1* ^*RC/RC*^	CAAAGGTCTGGGTGATAACTGGTG	CAGGACAGCCAAATAGACAGGG
*Pkd2* ^*fl/fl*^	GGGTGCTGAAGAGATGGTTC	TCCACAAAAGCTGCAATGAA
*Hnf1b* ^*fl/fl*^	GAGGACGGCGACGACTATGA	GGTGTCGTTAGTGAGATGCGT

### Cell culture and reagents

Murine IMCD3 cells were obtained from ATCC. Cells were maintained in DMEM supplemented with 10% FBS and 1% Penstrep (Invitrogen). For the generation of isogenic CRISPR/Cas9-mediated *Snhg5* knockouts, mIMCD3 cells were transfected with assembled ribonucleoprotein (RNP) complexes consisting of 1 μm recombinant Cas9 and two annealed Tracer/sgRNAs (IDT) in OptiMEM using RNAimax transfection reagent according to manufacturer’s specifications (Invitrogen). *Snhg5* sgRNA sequences are provided in Table 3. Media was changed the following day. After 48 h, single cells were sorted into 96-well plates using flow cytometry. Putative *Snhg5* knockout clones were screened using genomic PCR and confirmed with qRT-PCR. Primer sequences are provided in Table 2. For genotyping, genomic DNA was isolated from mouse tails using the NucleoSpin tissue genomic DNA kit (Macherey-Nagel). Nuclear and cytoplasmic fractionation was performed as previously described^23^.

**Table 3:** CRISPR SgRNA sequences.

Target Locus/Gene	sgRNA Sequence (5′–3′)	PAM
*Snhg5* gRNA1	AAAAGGAGCTGCGGTGAACC	GGG
*Snhg5* gRNA2	GAAGATTAACCAGAGTGCCC	TGG

### Animal studies

All animal experiments were performed in accordance with and under the auspices of the Institutional Animal Care and Use Committee at Stony Brook University or the University of Minnesota. *Pkd1*^fl/fl^^44^, *Pkd2*^fl/fl^^44^, *Hnf1-b*^fl/fl^^45^ and *Pkd1*^RC/RC^^26^ mice were used in this study. All experiments were performed on mice of both sexes on a C57Bl/6 background. *Sngh5*^−/−^ mice were generated at the Mouse Genetics Core at the University of Minnesota. In brief, two guide RNAs flanking the 1.9 kb *Snhg5* gene were annealed to a tracer RNA by incubating at 95 °C for 5 min and cooling to room temperature. Sequences for sgRNAs are provided in Table 3. RNPs, which contain annealed sgRNA/Tracer RNA and recombinant Cas9 enzyme (IDT), were prepared with 20 ng RNA and 80 ng Cas9 protein (IDT) and incubated at room temperature for 15 min to allow RNP complex formation. The mix was centrifuged at 13,000 RPM for 5 min, filtered through a Millipore filter, injected into C57BL/6J zygotes, and subsequently transferred into oviducts of pseudo-pregnant foster mothers. Founders with the desired genotype were identified by PCR and DNA sequencing and were bred to generate *Snhg5*^−/−^ mice.

### Western blotting

Western blotting was performed by lysing mIMCD3 cells in RIPA buffer (Sigma) supplemented with protease inhibitors for 10 min on ice. 20–30 ug of protein were loaded onto Nu-PAGE 4–12% Bis-Tris mini gels (Invitrogen), transferred to nitrocellulose and probed with anti-ARPC5 and b-actin (Sigma) antibodies. Proteins were detected using the Amersham ECL Western blot kit.

### RNA and qRT-PCR

Freshly harvested mouse kidneys were immediately transferred to Qiazol solution (Qiagen) on ice and homogenized with a mechanical grinder. Total RNA was extracted using the miRNeasy RNA purification kit (Qiagen) according to manufacturer’s specifications. cDNA was generated using the RevertAid cDNA synthesis kit (ThermoFisher), followed by quantitative real-time PCR with PerfeCTa SYBR Green SuperMix (Quanta bio). Reactions were run on the CFX Connect Real-Time System (Bio-Rad). Gene expression levels were normalized to b-actin (total) or U6 (nuclear). Primers for qPCR are listed in Table 1.

### RNA sequencing

Whole transcriptome RNA sequencing (RNA seq) was performed by Novogene Corp. (http://en.novogene.com). For bioinformatics, PCR and optical duplicates were removed from all fastq files with the bash Clumpify tool. Sorted BAM alignment files were generated with STAR using default settings. FeatureCounts was used for transcript quantification and annotation (gencode.vM36.chr_patch_hapl_scaff.annotation.gtf). Count files for both the in vivo and in vitro experiments were subsequently loaded into R (libraries in use: tidyverse, DESeq2, biomaRt, dplyr, purrr) and DESeq2 was employed (design = ~ condition + genotype) for obtaining differential gene calls. Significantly up and down-regulated genes were those with *p* value < 0.05 and log2 fold change differences above (upregulated) and below (downregulated) zero.

### Histology

Mice were euthanized by decapitation or by CO_2_ asphyxiation as appropriate for their age. Animals older than P14 were perfused with 20 ml PBS. Kidneys were excised, immersion fixed in ≥ 5 volumes of 4% paraformaldehyde for 48 h and transferred to 70% ethanol. Paraffin-embedded formalin-fixed 5 μm sections were stained with hematoxylin and eosin (H&E) or periodic acid–Schiff stain (PAS) for histology as previously described^25^.

### Creatinine measurement

During euthanasia, blood was collected from 14-day-old pups of the appropriate genetic background and placed into 1 ml blood collection serum gel microtubes (Sai Infusion Technologies) on ice. Samples were centrifuged 12,000 rpm for 5 min at room temperature, and serum stored at − 80 °C. Creatinine measurements were performed using Isotope Dilution LC-MSMS at the O’Brien Center for Acute Kidney Injury Research, University of Alabama at Birmingham (UAB-USCD).

### Cyst analysis

H&E-stained sections were digitally imaged using a scanning Nikon Eclipse Ni fluorescence microscope with a DS-Qi2 model camera. Quantification of cyst number (CN), cyst area, and total kidney area was performed using ImageJ (Fiji). Cysts were identified as dilations larger than three times the diameter of normal tubules. The Cyst Index (CI) was calculated using the following formula: (total cystic area/total kidney area) × 100. Both male and female animals were used in the analysis and scored independently and blindly by two investigators.

### Cell cycle analysis

Cell cycle distribution was assessed in wild-type and *Snhg5* knockout mIMCD3 cells using propidium iodide (PI) staining and flow cytometry. Cells were grown in six-well plates and trypsinized before full confluency. Cells were collected and centrifuged at 1500 rpm for 5 min, washed with cold PBS and fixed in 70% cold ethanol overnight. Cells were centrifuged at 1500 rpm for 5 min the following day and stained with 500 ml PI master mix (PI) (0.1% Triton X-100, 01mM EDTA disodium salt, 0.05 mg/ml RNase A (50unit/mg), 50 mg/ml PI, in PBS pH7.4) for one hour. PI cell cycle analysis was run on a Becton Dickinson LSRII with a 20mW Blue (488), 40mW Red (640), 25mW Violet (405) spatially separated laser sources. We used the 20mW Blue Laser and a 610/20 Band Pass Filter with a 600LP Filter to register the fluorescent signal. Doublet discrimination was used to analyze the cellular expression of PI via histogram and to collect a statistically relevant population percentage of single cells to analyze for the cell cycle profile. Cells were gated into three population phases based on PI fluorescence intensity: sub-G1/M1, corresponding to apoptotic or hypodiploid cells; G0/G1 (2 N DNA content); and combined S/G2/M (cells with intermediate to 4 N DNA content).

### RNAscope

In situ detection of *Snhg5* transcripts was performed using the RNAscope 2.5 HD Brown assay kit (Advanced Cell Diagnostics) according to the manufacturer’s protocol. Formalin-fixed, paraffin-embedded mouse kidney sections (5 μm) were used, including wild-type, *Pkd1*^*fl/fl*^ and *Hnf1b*^*fl/fl*^ cystic tissue. Briefly, slides were baked at 60 °C, deparaffinized with xylene, and treated with hydrogen peroxide. Sections underwent antigen retrieval at 95 °C for 15 min, and protease digestion for 15 min as detailed in the manufacturer’s instructions. Labeling and hybridization were done using a probe targeting mouse *Snhg5* (catalog 504501), and signal amplification was carried out using the RNAscope 2.5 HD series reagents. Chromogenic detection was achieved using DAB, and slides were counterstained with hematoxylin, dehydrated, and mounted on coverslips. Tissue sections were imaged using a scanning Nikon eclipse Ni fluorescence microscope with a DS-Qi2 model camera.

## Supplementary Information

Below is the link to the electronic supplementary material.

Supplementary Material 1

## Data Availability

The RNA-seq data generated in this study have been deposited in the GEO/SRA database under accession code GSE300255. The remaining data and information are available within the article, supplementary information or available from Dr. Karam Aboudehen (karam.aboudehen@Stonybrookmedicine.edu) upon request.

## References

[1] Mattick, J. S. et al. Long non-coding RNAs: Definitions, functions, challenges and recommendations. *Nat. Rev. Mol. Cell. Biol.* **24**, 430–447. 10.1038/s41580-022-00566-8 (2023). 36596869 10.1038/s41580-022-00566-8 PMC10213152

[2] Derrien, T. et al. The GENCODE v7 catalog of human long noncoding rnas: Analysis of their gene structure, evolution, and expression. *Genome Res.* **22**, 1775–1789. 10.1101/gr.132159.111 (2012). 22955988 10.1101/gr.132159.111 PMC3431493

[3] Rinn, J. L. & Chang, H. Y. Genome regulation by long noncoding RNAs. *Annu. Rev. Biochem.* **81**, 145–166. 10.1146/annurev-biochem-051410-092902 (2012). 22663078 10.1146/annurev-biochem-051410-092902 PMC3858397

[4] Fatica, A. & Bozzoni, I. Long non-coding RNAs: New players in cell differentiation and development. *Nat. Rev. Genet.* **15**, 7–21. 10.1038/nrg3606 (2014). 24296535 10.1038/nrg3606

[5] Statello, L., Guo, C. J., Chen, L. L. & Huarte, M. Author correction: gene regulation by long non-coding RNAs and its biological functions. *Nat. Rev. Mol. Cell. Biol.* **22**, 159. 10.1038/s41580-021-00330-4 (2021). 33420484 10.1038/s41580-021-00330-4 PMC8095262

[6] Chi, Y., Wang, D., Wang, J., Yu, W. & Yang, J. Long non-coding RNA in the pathogenesis of cancers. *Cells*10.3390/cells8091015 (2019). 31480503 10.3390/cells8091015 PMC6770362

[7] Cui, M. et al. Long non-coding RNA PVT1 and cancer. *Biochem. Biophys. Res. Commun.* **471**, 10–14. 10.1016/j.bbrc.2015.12.101 (2016). 26850852 10.1016/j.bbrc.2015.12.101

[8] Ghetti, M., Vannini, I., Storlazzi, C. T., Martinelli, G. & Simonetti, G. Linear and circular PVT1 in hematological malignancies and immune response: Two faces of the same coin. *Mol. Cancer*. **19**, 69. 10.1186/s12943-020-01187-5 (2020). 32228602 10.1186/s12943-020-01187-5 PMC7104523

[9] Wu, F. et al. Regulation mechanism and pathogenic role of LncRNA plasmacytoma variant translocation 1 (PVT1) in human diseases. *Genes Dis.* **10**, 901–914. 10.1016/j.gendis.2022.05.037 (2023). 37396533 10.1016/j.gendis.2022.05.037 PMC10308122

[10] Hou, J., Zhang, G., Wang, X., Wang, Y. & Wang, K. Functions and mechanisms of LncRNA MALAT1 in cancer chemotherapy resistance. *Biomark. Res.* **11**, 23. 10.1186/s40364-023-00467-8 (2023). 36829256 10.1186/s40364-023-00467-8 PMC9960193

[11] Han, W., Shi, J., Cao, J. C., Dong, B. & Guan, D. W. Latest advances of long non-coding RNA SNHG5 in human cancers. *Oncotargets Ther.* **13**, 6393–6403. 10.2147/Ott.S252750 (2020). 10.2147/OTT.S252750 PMC7342554 32753882

[12] Hao, J. et al. Lnc-SNHG5 promoted hepatocellular carcinoma progression through the RPS3-NFκB pathway. *Int. J. Gen. Med.* **16**, 5651–5664. 10.2147/Ijgm.S442937 (2023). 38059157 10.2147/IJGM.S442937 PMC10697148

[13] Li, X. et al. Long non-coding RNA SNHG5 promotes glioma progression via miR-205/E2F3 axis. *Biosci. Rep.*10.1042/BSR20190668 (2019). 31292168 10.1042/BSR20190668 PMC6639464

[14] Li, Y. R. et al. Long non-coding RNA SNHG5 promotes human hepatocellular carcinoma progression by regulating miR-26a-5p/GSK3β signal pathway. *Cell Death Dis* **9**, doi:ARTN 88810.1038/s41419-018-0882-5 (2018). 10.1038/s41419-018-0882-5 PMC6117363 30166525

[15] Meng, X. et al. LncRNA SNHG5 promotes proliferation of glioma by regulating miR-205-5p/ZEB2 axis. *Onco Targets Ther.* **12**, 11487–11496. 10.2147/OTT.S228439 (2019). 31920337 10.2147/OTT.S228439 PMC6939796

[16] Zhang, M. B. et al. LncRNA SNHG5 affects cell proliferation, metastasis and migration of colorectal cancer through regulating miR-132-3p/. *Cancer Biol. Ther.* **20**, 524–536. 10.1080/15384047.2018.1537579 (2019). 30395767 10.1080/15384047.2018.1537579 PMC6422517

[17] Zhou, Y. et al. Long noncoding RNA SNHG5 promotes podocyte injury via the microRNA-26a-5p/TRPC6 pathway in diabetic nephropathy. *J. Biol. Chem.* **298**, doi:ARTN 10260510.1016/j.jbc.102605 (2022). (2022). 10.1016/j.jbc.2022.102605 PMC9694110 36257404

[18] Wang, M., Wei, J. L., Shang, F. T., Zang, K. & Zhang, P. Down-regulation of LncRNA SNHG5 relieves sepsis-induced acute kidney injury by regulating the miR-374a-3p/TLR4/NF-κB pathway. *J. Biochem.* **169**, 575–583. 10.1093/jb/mvab008 (2021). 33479745 10.1093/jb/mvab008

[19] Wilson, P. D. Polycystic kidney disease: New understanding in the pathogenesis. *Int. J. Biochem. Cell. B*. **36**, 1868–1873. 10.1016/j.biocel.2004.03.012 (2004). 10.1016/j.biocel.2004.03.012 15203099

[20] Lanktree, M. B. et al. Prevalence estimates of polycystic kidney and liver disease by population sequencing. *J. Am. Soc. Nephrol.* **29**, 2593–2600. 10.1681/Asn.2018050493 (2018). 30135240 10.1681/ASN.2018050493 PMC6171271

[21] Harris, P. C. & Torres, V. E. Polycystic kidney disease. *Annu. Rev. Med.* **60**, 321–337. 10.1146/annurev.med.60.101707.125712 (2009). 18947299 10.1146/annurev.med.60.101707.125712 PMC2834200

[22] Hateboer, N. et al. Comparison of phenotypes of polycystic kidney disease types 1 and 2. *Lancet* 353, 103–107 10.1016/S0140-6736(98)03495-3 (1999). 10.1016/s0140-6736(98)03495-3 10023895

[23] Aboudehen, K. et al. Long noncoding RNA is dysregulated in autosomal dominant polycystic kidney disease and regulates mTOR signaling. *J. Biol. Chem.* **293**, 9388–9398. 10.1074/jbc.RA118.001723 (2018). 29716997 10.1074/jbc.RA118.001723 PMC6005429

[24] Eckberg, K. et al. Small hairpin inhibitory RNA delivery in the metanephric organ culture identifies long noncoding RNA as a modulator of cyst growth. *Am. J. Physiol. Renal Physiol.* **323**, F335–F348. 10.1152/ajprenal.00016.2022 (2022). 10.1152/ajprenal.00016.2022 PMC9423782 35862648

[25] Weisser, I., Eckberg, K., D’Amico, S., Buttram, D. & Aboudehen, K. Ablation of long noncoding RNA exacerbates cystogenesis in mouse polycystic kidney disease. *J. Am. Soc. Nephrol.* **35**, 41–55 10.1681/Asn.0000000000000265 (2024). 37953472 10.1681/ASN.0000000000000265 PMC10786614

[26] Hopp, K. et al. Functional polycystin-1 dosage governs autosomal dominant polycystic kidney disease severity. *J. Clin. Invest.* **122**, 4257–4273. 10.1172/Jci64313 (2012). 23064367 10.1172/JCI64313 PMC3484456

[27] Li, Y. H., Hu, Y. Q., Wang, S. C., Li, Y. & Chen, D. M. LncRNA SNHG5: A new budding star in human cancers. *Gene* **749** ARTN 14472410.1016/j.gene.2020.144724 (2020). 10.1016/j.gene.2020.144724 32360843

[28] Muto, Y. et al. Defining cellular complexity in human autosomal dominant polycystic kidney disease by multimodal single cell analysis. *Nature Commun.* **13**, doi:ARTN 649710.1038/s41467-022-34255-z (2022). 10.1038/s41467-022-34255-z PMC9618568 36310237

[29] Haarer, E. L., Theodore, C. J., Guo, S., Frier, R. B. & Campellone, K. G. Genomic instability caused by Arp2/3 complex inactivation results in micronucleus biogenesis and cellular senescence. *PLoS Genet.* **19**, e1010045. 10.1371/journal.pgen.1010045 (2023). 36706133 10.1371/journal.pgen.1010045 PMC9907832

[30] Moulding, D. A. et al. Excess F-actin mechanically impedes mitosis leading to cytokinesis failure in X-linked neutropenia by exceeding Aurora B kinase error correction capacity. *Blood* **120**, 3803–3811. 10.1182/blood-2012-03-419663 (2012). 22972986 10.1182/blood-2012-03-419663 PMC4338607

[31] Sadhu, L. et al. ARPC5 isoforms and their regulation by calcium-calmodulin-N-WASP drive distinct Arp2/3-dependent actin remodeling events in CD4 T cells. *Elife*10.7554/eLife.82450 (2023). 10.7554/eLife.82450 PMC10171864 37162507

[32] Silverman-Gavrila, R. et al. Rear polarization of the microtubule-organizing center in neointimal smooth muscle cells depends on PKCalpha, ARPC5, and RHAMM. *Am. J. Pathol.* **178**, 895–910. 10.1016/j.ajpath.2010.10.001 (2011). 21281821 10.1016/j.ajpath.2010.10.001 PMC3128507

[33] Sun, S. C. et al. Arp2/3 complex regulates asymmetric division and cytokinesis in mouse oocytes. *PLoS One*. **6**, e18392. 10.1371/journal.pone.0018392 (2011). 21494665 10.1371/journal.pone.0018392 PMC3072972

[34] Patel, V. et al. Acute kidney injury and aberrant planar cell Polarity induce cyst formation in mice lacking renal cilia. *Hum. Mol. Genet.* **17**, 1578–1590. 10.1093/hmg/ddn045 (2008). 18263895 10.1093/hmg/ddn045 PMC3150596

[35] Cabili, M. N. et al. Integrative annotation of human large intergenic noncoding RNAs reveals global properties and specific subclasses. *Genes Dev.* **25**, 1915–1927. 10.1101/gad.17446611 (2011). 21890647 10.1101/gad.17446611 PMC3185964

[36] Statello, L., Guo, C. J., Chen, L. L. & Huarte, M. Gene regulation by long non-coding RNAs and its biological functions. *Nat. Rev. Mol. Cell. Biol.* **22**, 96–118. 10.1038/s41580-020-00315-9 (2021). 33353982 10.1038/s41580-020-00315-9 PMC7754182

[37] Lee, E. J. Cell proliferation and apoptosis in ADPKD. *Cystogenesis* **933**, 25–34 10.1007/978-981-10-2041-4_3 (2016). 10.1007/978-981-10-2041-4_3 27730432

[38] Sauvageau, M. et al. Multiple knockout mouse models reveal LincRNAs are required for life and brain development. *Elife* **2** doi:ARTN e0174910.7554/eLife.01749. (2013). 10.7554/eLife.01749 PMC3874104 24381249

[39] Damas, N. D. et al. promotes colorectal cancer cell survival by counteracting STAU1-mediated mRNA destabilization. *Nature Commun.* **7** doi:ARTN 1387510.1038/ncomms13875 (2016). 10.1038/ncomms13875 PMC5192221 28004750

[40] Liu, L. M. et al. si-SNHG5-FOXF2 inhibits TGF-β1-induced fibrosis in human primary endometrial stromal cells by the Wnt/β-catenin signalling pathway. *Stem Cell Res Ther* **11** doi:ARTN 47910.1186/s13287-020-01990-3 (2020). 10.1186/s13287-020-01990-3 PMC7656702 33176855

[41] Chebib, F. T. & Torres, V. E. Recent advances in the management of autosomal dominant polycystic kidney disease. *Clin. J. Am. Soc. Nephro*. **13**, 1765–1776. 10.2215/Cjn.03960318 (2018). 10.2215/CJN.03960318 PMC6237066 30049849

[42] Li, Q. et al. Heterogeneity of cell composition and origin identified by single-cell transcriptomics in renal cysts of patients with autosomal dominant polycystic kidney disease. *Theranostics* **11**, 10064–10073. 10.7150/thno.57220 (2021). 34815804 10.7150/thno.57220 PMC8581434

[43] Goff, L. A. & Rinn, J. L. Linking RNA biology to LncRNAs. *Genome Res.* **25**, 1456–1465. 10.1101/gr.191122.115 (2015). 26430155 10.1101/gr.191122.115 PMC4579330

[44] Shibazaki, S. et al. Cyst formation and activation of the extracellular regulated kinase pathway after kidney specific inactivation of. *Hum. Mol. Genet.* **17**, 1505–1516. 10.1093/hmg/ddn039 (2008). 18263604 10.1093/hmg/ddn039 PMC2902289

[45] Pontoglio, M. et al. Defective insulin secretion in hepatocyte nuclear factor 1α-deficient mice. *J. Clin. Invest.* **101**, 2215–2222 (1998). doi:Doi 10.1172/Jci2548. 9593777 10.1172/JCI2548 PMC508809

