# Supplementary material for: Loss of Snhg5 disrupts cell-cycle regulation without altering cystogenesis in a mouse model of polycystic kidney disease
Source: Sci Rep. 2026 Jan 8;16:4869. doi: 10.1038/s41598-026-35234-w (PMC12873420; doi:10.1038/s41598-026-35234-w)
Supplement: Supplementary file 1 — Supplementary Material 1 [file 41598_2026_35234_MOESM1_ESM.pdf]

Loss of *Snhg5* disrupts cell-cycle regulation without altering cystogenesis in a mouse model of polycystic kidney disease.

Stephen D'Amico[1+], Ujala Dar[1+], Kara Eckberg[2], Ivan Weisser[2], Chandrema Hossain[1], Robert Bronstein[1], and Karam Aboudehen[1,2*]

[1] Division of Nephrology & Hypertension, Department of Medicine, Stony Brook University, Stony Brook, NY, USA

[2] Department of Medicine, University of Minnesota, Minneapolis, Minnesota, USA

+These authors contributed equally to this work.

*Correspondence should be addressed to Karam Aboudehen (karam.aboudehen@stonybrookmedicine.edu).

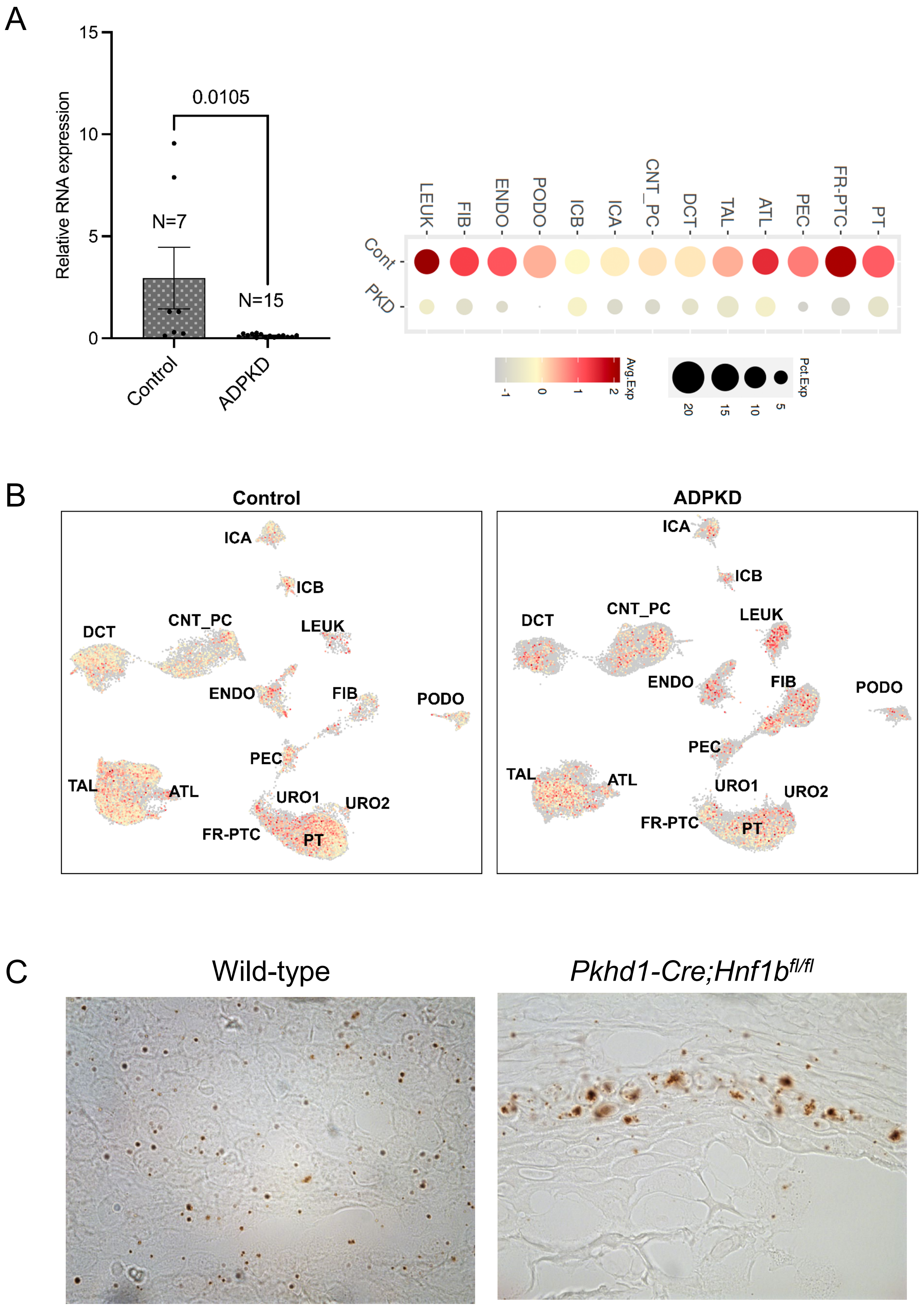

Supplemental figure S1

Supplemental figure S1. *Snhg5/SNHG5* expression in the human and mouse A) qRT-PCR measuring the average expression of *SNHG5* in nephrectomy specimens from patients with ADPKD (n=15) compared to matched normal controls (n=7). Error bars represent SD. **B)** UMAP showing density-based clustering from single-nuclear RNA-seq (snRNA-seq) of normal human kidneys (control) and kidneys from patients with ADPKD. **C)** RNAscope demonstrating the localization of *Snhg5* RNA in the kidneys of wild-type (left), *Pkhd1*-Cre; *Hnf1b*$^{fl/fl}$ (right) mice.

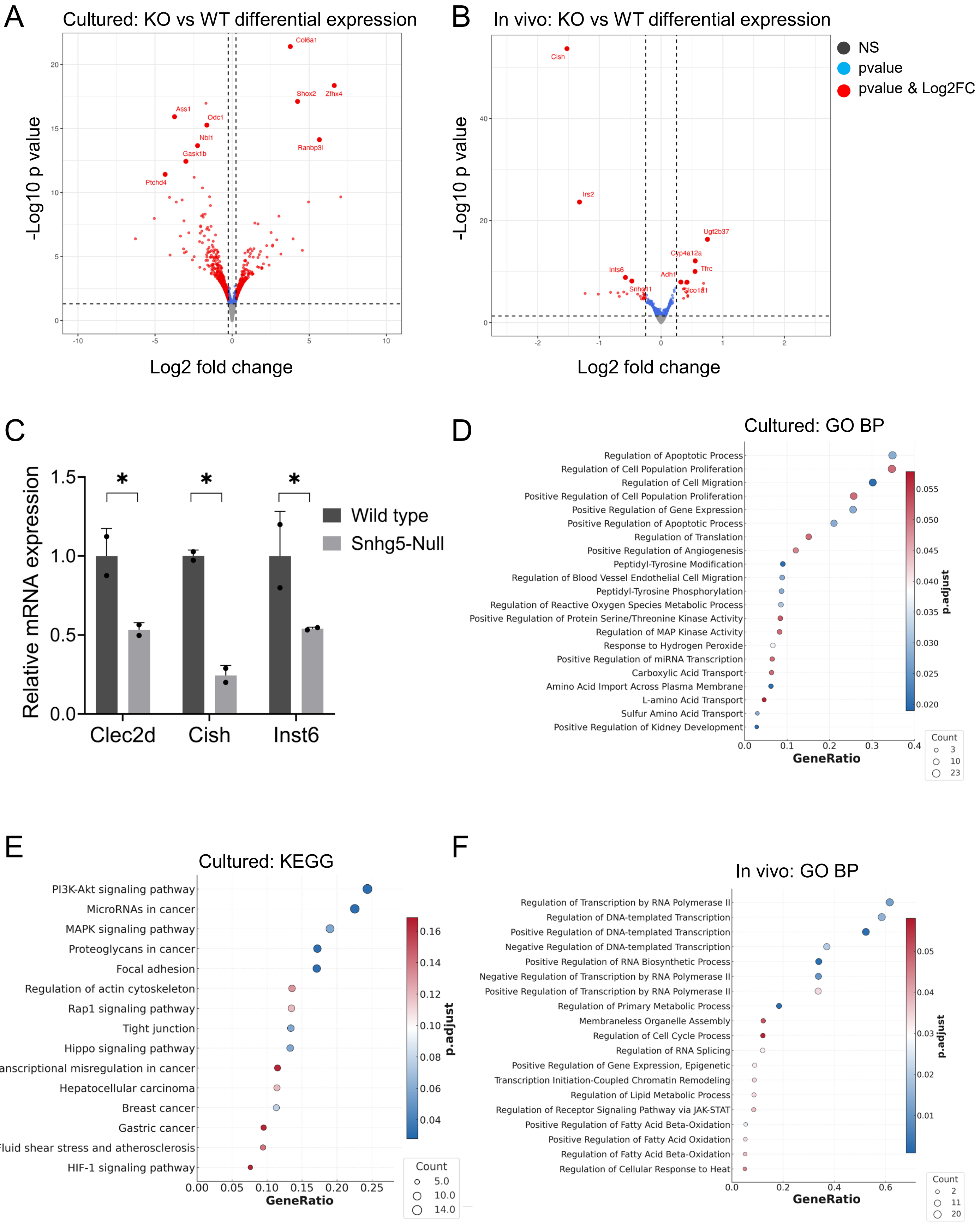

Supplemental figure S2

Supplemental figure S2. In vitro and in vivo RNA-seq A-B. Volcano plots depicting all differentially expressed genes in **A)** isogenic wild-type vs *Snhg5*-null mIMCD3 cell lines and **B)** wild-type vs *Snhg5*-null mouse kidneys. **C)** qRT-PCR validation of select genes from independent *Snhg5*-null kidneys relative to wild-type controls (n=2). Asterisks denote $p<0.05$. **D)** Gene ontology (biological process) analysis derived from RNA seq of wild-type and *Snhg5*-null mIMCD3 cells. **E)** Pathways analysis (KEGG) derived from RNA seq of wild-type and *Snhg5*-null mIMCD3 cells. **F)** Gene ontology (biological process) analysis derived from RNA seq of wild-type and *Snhg5*-null mouse kidneys.

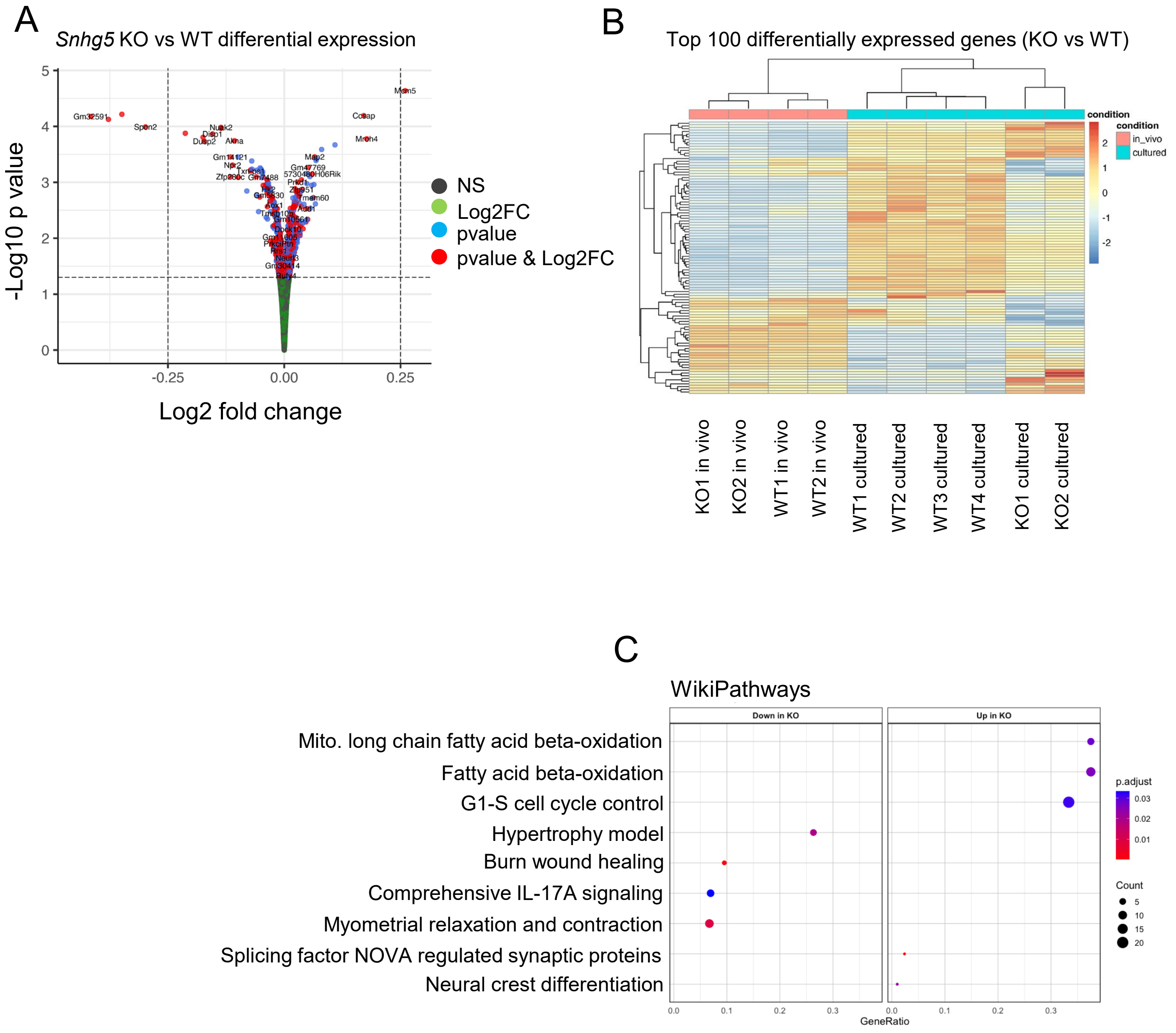

Supplemental figure S3

Supplemental figure S3. Integrative RNA seq A) Volcano plot depicting all differentially expressed genes shared between *Snhg5*-null mIMCD3 cells and mouse kidneys. **B)** Unsupervised hierarchical clustering of the top 100 genes ($p<0.05$) derived from integrative RNA seq of *Snhg5*-intact (n=6) and null (n=4) mIMCD3 cells and kidneys. Rows represent differentially expressed genes. Columns represent biological replicates (n= 10). Expression values are shown as Z-scores of normalized CPM, with red indicating higher and blue indicating lower expression relative to the mean across samples. **C)** Pathway analysis (WikiPathways) displaying the top altered pathways in *Snhg5*-intact and null mIMCD3 cells and kidneys.

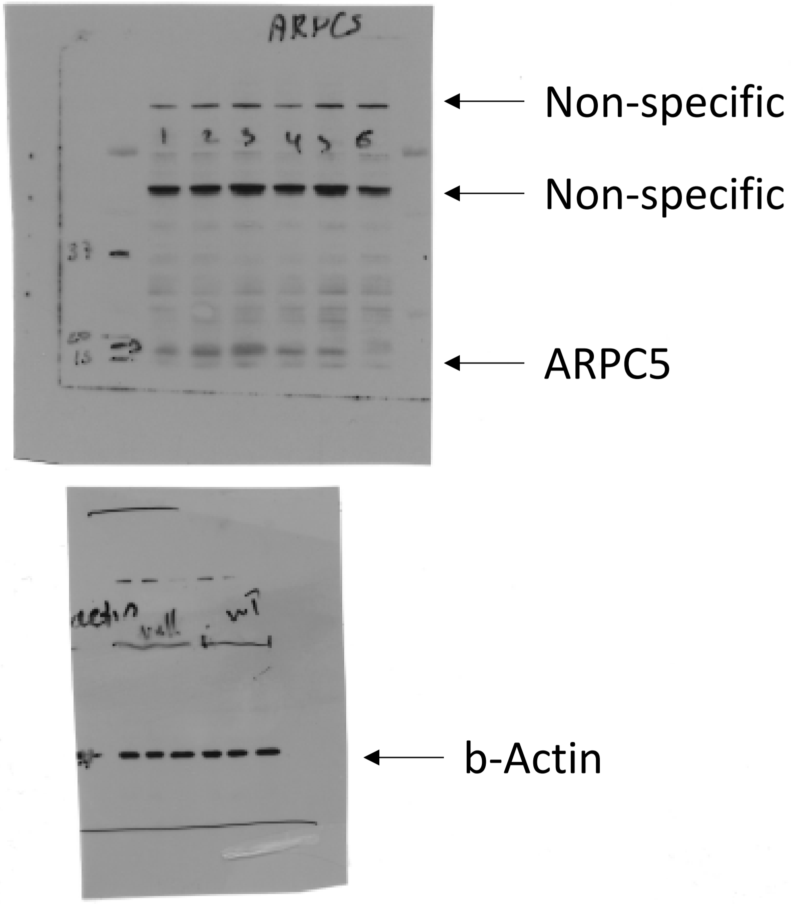

Uncropped Western blots
